# *Rnf138* deficiency promotes apoptosis of spermatogonia in juvenile male mice

**DOI:** 10.1038/cddis.2017.110

**Published:** 2017-05-18

**Authors:** Longchang Xu, Yalan Lu, Deqiang Han, Rongyan Yao, Han Wang, Shunshun Zhong, Yanyun Luo, Ruiqin Han, Kai Li, Jun Fu, Shudong Zong, Shiying Miao, Wei Song, Linfang Wang

**Affiliations:** 1 Department of Biochemistry and Molecular Biology, State Key Laboratory of Medical Molecular Biology, Institute of Basic Medical Sciences Chinese Academy of Medical Sciences, Peking Union Medical College, Beijing 100005, China; 2 National Research Institute for Family Planning, WHO Collaboration Center of Human Reproduction, Da Hui Si Road, Beijing 100081, China

## Abstract

Spermatogenesis, the process by which haploid sperm cells are produced from a diploid precursor cell, is essential for sexual reproduction. Here, we report that RING-finger protein 138 (*Rnf138*) is highly expressed in testes, especially in spermatogonia and spermatocytes. The role of *Rnf138* in spermatogenesis was examined using a *Rnf138*-knockout mouse model. *Rnf138* deficiency resulted in increased apoptosis in spermatogenic cells, loss of proliferative spermatogonia, delayed development of spermatozoa and impaired fertility. The proportion of PLZF+Ki67+ cells within the PLZF+ population decreased in the knockout mice. The phenotype was further assessed by RNA-sequencing (RNA-seq), which determined that the expression levels of many genes involved in spermatogenesis were altered in the testis of *Rnf138*-knockout mice. Thus, *Rnf138* deficiency promotes the apoptosis of spermatogenic cells, which may have been caused by the aberrant proliferation of spermatogonia in mouse testis development.

Spermatogenesis is a complex process comprised of sequential mitotic, meiotic and spermiogenic phases.^1^ The proliferative phase is carried out by undifferentiated and differentiating spermatogonia. Proliferation is initiated by type A_single_ spermatogonia, which give rise to type A_paired_ followed by type A_aligned_ spermatogonia, collectively referred to as undifferentiated spermatogonia. Differentiation begins when A_aligned_ transform into A_1_ spermatogonia. The differentiating spermatogonia progress from A_1_ to A_2_, A_3_, A_4_, In and B spermatogonia in six rounds of cell division, and finally transform into meiotic spermatocytes.^2, 3, 4, 5^ Meiosis is a unique form of cell division taking place exclusively in reproductive organs, which generates haploid gametes from diploid cells. The mechanisms and regulation of meiosis are similar to mitosis but differ in vital processes such as cell-cycle regulation, recombination and chromosome segregation. During meiosis, the genetic material can be exchanged between two homologous chromosomes via meiotic recombination with the formation of the synaptonemal complex.^6^ After two rounds of cell division, spermatogenesis moves into the spermiogenic phase during which round spermatids mature into elongated spermatids.^5^

Ubiquitination has important roles in the maintenance of spermatogonial stem cells, meiosis and spermiogenesis.^7^ The regulatory functions of ubiquitination vary greatly depending on the site of ubiquitination and differences in ubiquitin chain forms.^8, 9, 10^ The testes are ubiquitin-ligating (E3) enzyme-rich organs containing significantly more specialized E3 enzymes compared with other tissues, and several E3 ligases with key functions in spermatogenesis have previously been identified.^11, 12^

The gene encoding the RING-finger protein 138 (RNF138), also known as HSD-4 or NARF, was first isolated in our lab from a human testis-subtracted cDNA library in 1999 (GenBank: AF162680.3). It contains an N-terminal C3HC4 (Cys(3)-His-Cys(4)) RING domain, three zinc-finger-like domains and a C-terminal UIM-type (ubiquitin-interacting motif) domain.^13^ RNF138 has been shown to function as an E3 ligase in the regulation of Wnt-*β*-catenin signaling by interacting with the negative regulator NLK to target the Wnt-activating cofactor TCF/LEF for degradation by ubiquitination.^14^ Furthermore, two recent studies discovered that RNF138 is a key homologous recombination (HR)-promoting factor that regulates DNA repair by displacing Ku and ubiquitinating CtIP.^15, 16^ Even though *Rnf138* was first identified in testes, its role in spermatogenesis has not yet been studied. Here, we generated *Rnf138*-knockout mice and determined the localization of RNF138 in the seminiferous tubule. Strikingly, we found that knockout of *Rnf138* delayed spermatogenesis in juvenile males and significantly impaired their fertility, as indicated by the decreased proliferative spermatogonia and sperm counts. We therefore discussed the role of *Rnf138* in regulating the apoptosis of proliferative spermatogonia.

## Results

### Temporospatial expression pattern of *Rnf138* in mice

**Figure 1:**
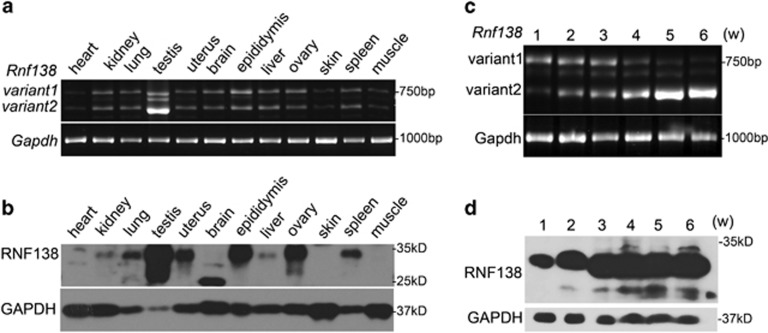
Temporo-patial expression pattern of *Rnf138* in mice. (**a**) RT-PCR analysis of the *Rnf138* mRNA transcription in 12 tissues, two variants (variants 1 and 2) were detected. (**b**) Western blot analysis of the expression of RNF138 in 12 tissues. (**c**) RT-PCR analysis of the *Rnf138* mRNA transcription in developing mouse testes during weeks 1 to 6 in testes. (**d**) Western blot analysis of the RNF138 protein expression in testes during weeks 1 to 6. Levels of glyceraldehyde 3-phosphate dehydrogenase (*Gapdh*) DNA were used as a loading control in all the RT-PCR analysis and GAPDH serves as a control in all the western blot assays. w, weeks

The tissue-specific expression pattern of *Rnf138* was determined by reverse transcription-PCR (RT-PCR) using a primer pair spanning the full length of the *Rnf138* coding sequence. As shown in Figure 1a, two variants (variants 1 and 2) of *Rnf138* were detected in all 12 tissues examined, with highest expression in the testis. The sequences of the two bands corresponding to isoform 1 (NM_207623.2) and isoform 2 (NM_019706.3) of *Rnf138* in the GenBank were confirmed by sequencing. In concordance with the transcript distribution, low levels of RNF138 protein were detected by western blot in multiple tissues, including kidney, lung, uterus, epididymis, liver, ovary and spleen, but protein expression was high in testis (Figure 1b).

Then, the expression pattern of *Rnf138* in testis was examined. During the first 6 weeks development, the two *Rnf138* transcript variants followed different expression patterns in the testis (Figure 1c). Expression of variant 1 was high during the first 3 weeks and began to decrease after week 4. Variant 2, however, was only weakly expressed at week 1 but increased every week throughout the testis development. These expression profiles indicate that these two variants may have different regulatory functions at different phases of testis development. The RNF138 protein product was detectable as early as week 1, and protein levels increased with age (Figure 1d). The high expression and the specific regulation pattern of *Rnf138* in testis indicate that Rnf138 may have a role during the testis development. The variant 1 and variant 2 could be restricted to a small population in the testis.

### Generation of *Rnf138*-knockout mice

**Figure 2:**
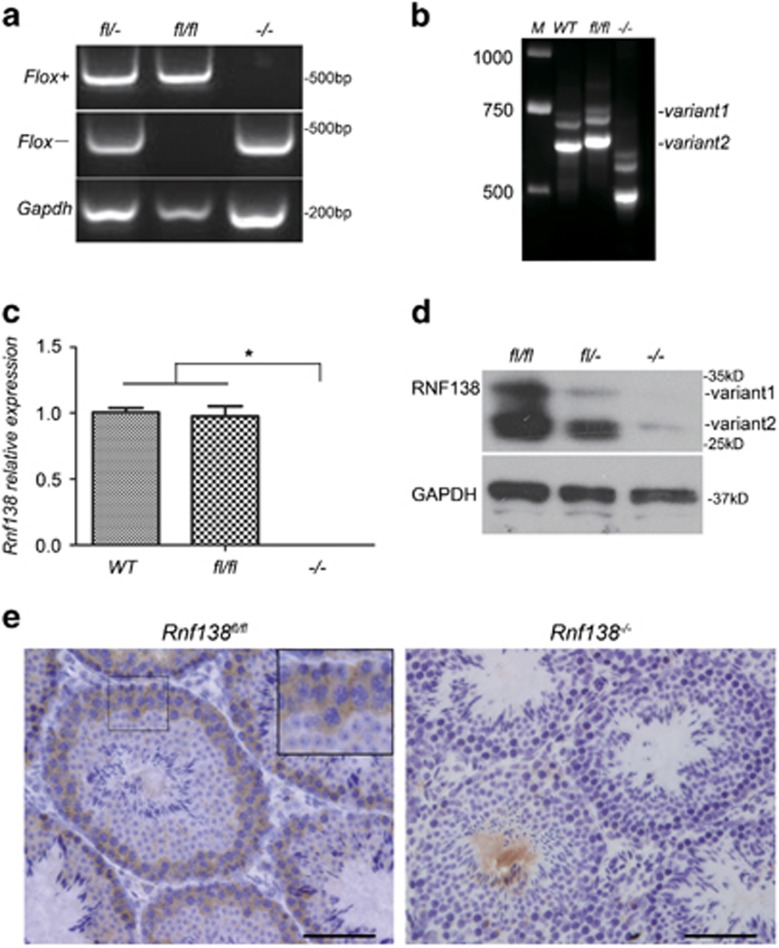
Generation of *Rnf138-*knockout mice. (**a**) PCR genotyping examined the *lox*P site (flox+) and the recombination deleted exon 2 of *Rnf138* (flox−) using DNA isolated from tails snips. Bands of both Flox+ and Flox− can be seen in *fl/*− Only the band of Flox+ can be seen in *fl/fl*;^−/−^ has one band of Flox− only. (**b**) RT-PCR identified the deletion of exon 2 in testis of *Rnf138*^*−/−*^. Two variants of *Rnf138* (variants 1 and 2) were detected. The deletion of exon 2 resulted in a shorter band in the column of −/−. (**c**) Real-time PCR analysis of the mRNA transcription of *Rnf138* in genotype *Rnf138*^*WT*^, *Rnf138*^*fl/fl*^ and *Rnf138*^*−/−*^ testes. Results were normalized against *actin* and expressed as mean±S.D. of three independent experiments (*P<0.05). (**d**) Western blot detected the expression of RNF138 in testes from *Rnf138*^*fl/fl*^, *Rnf138*^*fl/−*^ and *Rnf138*^*−/−*^. Representative plots of three independent experiments were shown. (**e**) Immunohistochemical analysis of the localizaton of RNF138 in mice testis. Pictorial data showed representatives of three independent experiments. The area shown with black dotted square was magnified in the upper-right corner. The bar represented 50 *μ*m. *fl/fl*, *fl/−*, *−/−* represent *Rnf138*^*fl/fl*^, *Rnf138*^*fl/−*^ and *Rnf138*^*−/−*^, respectively. M, marker; WT, wild type

To avoid embryonic death, female *Rnf138*^*fl/fl*^ mice were first crossed with males carrying the *Vasa-Cre* transgene. The resulting male heterozygous mice with the *Rnf138*^*+/fl*^*^/−^Vasa-Cre* genotype were then backcrossed to female *Rnf138*^*fl/fl*^ mice. Resulting offspring with *Rnf138*^*fl/−*^ genotype without the *Vasa-Cre* transgene were used to generate the homozygous *Rnf138*-knockout strain (*Rnf138*^*−/−*^) to eliminate any potential effects of the *Cre* transgene (Supplementary Figure 1sA). *Rnf138*^*fl/fl*^ and littermate *Rnf138*^*−/−*^ were obtained from *Rnf138*^*fl/−*^ mating with the same genotype. Age-matched *Rnf138*^*fl/fl*^ were used as controls. The genotype was determined by PCR using genomic DNA isolated from tail snips as template (Figure 2a). The correct transcript size was further confirmed by testis RT-PCR, where the exon 2 deletion resulted in a truncated transcript (Figure 2b), and by quantitative real-time PCR (qRT-PCR), where the knockout transcript could not be detected by exon 2-specific primers (Figure 2c). Note that this exon 2 deletion resulted in a downstream frameshift.

*Rnf138*-knockout (*Rnf138*^*−*/*−*^) mice were born alive and appeared anatomically normal. The number of homozygous and heterozygous mice showed normal Mendelian frequencies, suggesting that *Rnf138* may not be necessary for the embryonic development of mice. Deletion of the RNF138 protein was confirmed by western blot analysis with our RNF138 antibody, the specificity of which was pretested using *Rnf138* knockdown or knockout in spermatogonium GC1-spg and spermatocyte GC2-spd cells generated by siRNA or CRISPR/Cas9 (Supplementary Figures 2sA and B). As expected, RNF138 bands (variants 1 and 2) were absent from the testes harvested from *Rnf138*^*−/−*^ mice (Figure 2d).

To locate RNF138 expression in testis, immunohistochemical staining was used. By comparing the testes staining between *Rnf138*^*fl/fl*^ and *Rnf138*^*−/−*^ at 7 weeks, the results showed that RNF138 was mainly expressed in the spermatogonia and spermatocytes (Figure 2e). In conclusion, we successfully generated viable *Rnf138*-knockout mice and determined the localization of RNF138 in mouse testis.

### *Rnf138*-knockout impairs testis development

**Figure 3:**
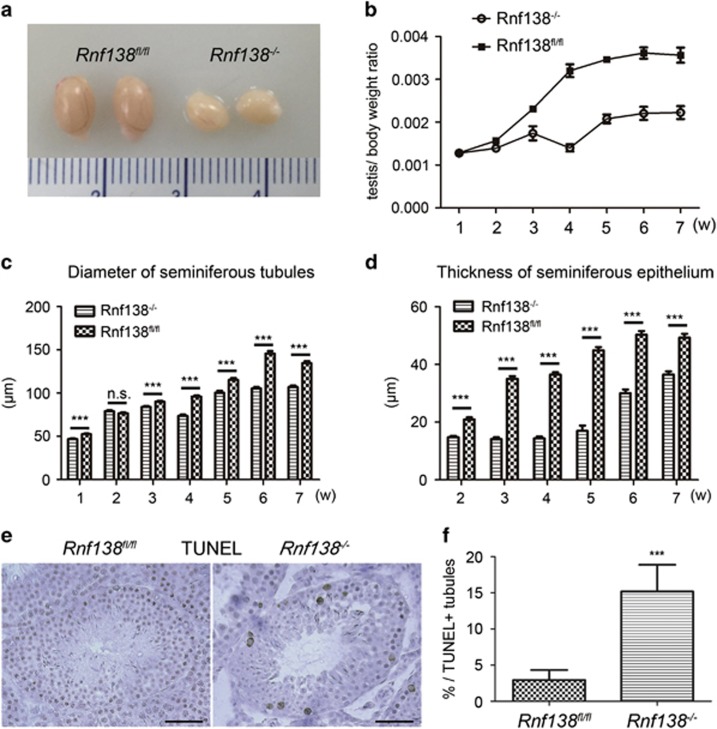
*Rnf138*-knockout impairs testis development. (**a**) Testes harvested from *Rnf138*^*fl/fl*^ and *Rnf138*^*−/−*^ at 7 weeks of age. (**b**) Testis/body weight ratio of *Rnf138*^*fl/fl*^ and *Rnf138*^*−/−*^ during weeks 1 to 7 (*n*=3). (**c** and **d**) The diameter and thickness of seminiferous tubules (*n*=40) in *Rnf138*^*fl/fl*^ and *Rnf138*^*−/−*^ during weeks 1 to 7. (**e**) TUNEL assay to detect apoptotic cells in the testes of *Rnf138*^*fl/fl*^ and *Rnf138*^*−/−*^ mice at 6 weeks of age. (**f**) Quantification and comparison of apoptotic cells in the seminiferous tubules between the control and *Rnf138*^*−/−*^ at 6 weeks (*n*=3). Values are mean±S.D. ***P<0.001; NS, no significance

To next evaluate the effect of *Rnf138* on the development of mouse testes, testes of different ages were harvested. Testes isolated from male *Rnf138*^*−*/*−*^ mice older than 2 weeks were significantly smaller than those from age-matched *Rnf138*^*fl/fl*^ mice, and by adult age (7 weeks), control testes weighed nearly two times as much as *Rnf138*^*−/−*^testes (Figures 3a and b). To determine the underlying causes of this testicular atrophy, the diameter of the seminiferous tubule and the thickness of the seminiferous epithelium were examined during weeks 1 through 7. The seminiferous tubule in *Rnf138*^*−/−*^ mice was significantly smaller than in control mice, except at 2 weeks of age (Figure 3c). In addition, the seminiferous epithelium was up to 50% thinner in knockout mice compared with control mice older than 2 weeks of age (Figure 3d). To further test if apoptosis contributed to the testicular atrophy, terminal deoxynucleotidyl transferase dUTP nick-end labeling (TUNEL) was used and the result showed significant increase of the apoptotic index in *Rnf138*^*−/−*^ testes at 6 weeks (Figure 3e). Quantification of the TUNEL assay clearly showed significant increase in the percent of TUNEL-positive tubules in the *Rnf138*^*−/−*^ testes, where ~15% tubules were TUNEL positive (Figure 3f). In summary, *Rnf138*, which prevents the apoptosis of spermatogenic cells, is required for the testes development.

### Deletion of *Rnf138* results in mildly delayed spermatogenesis in juvenile males

**Figure 4:**
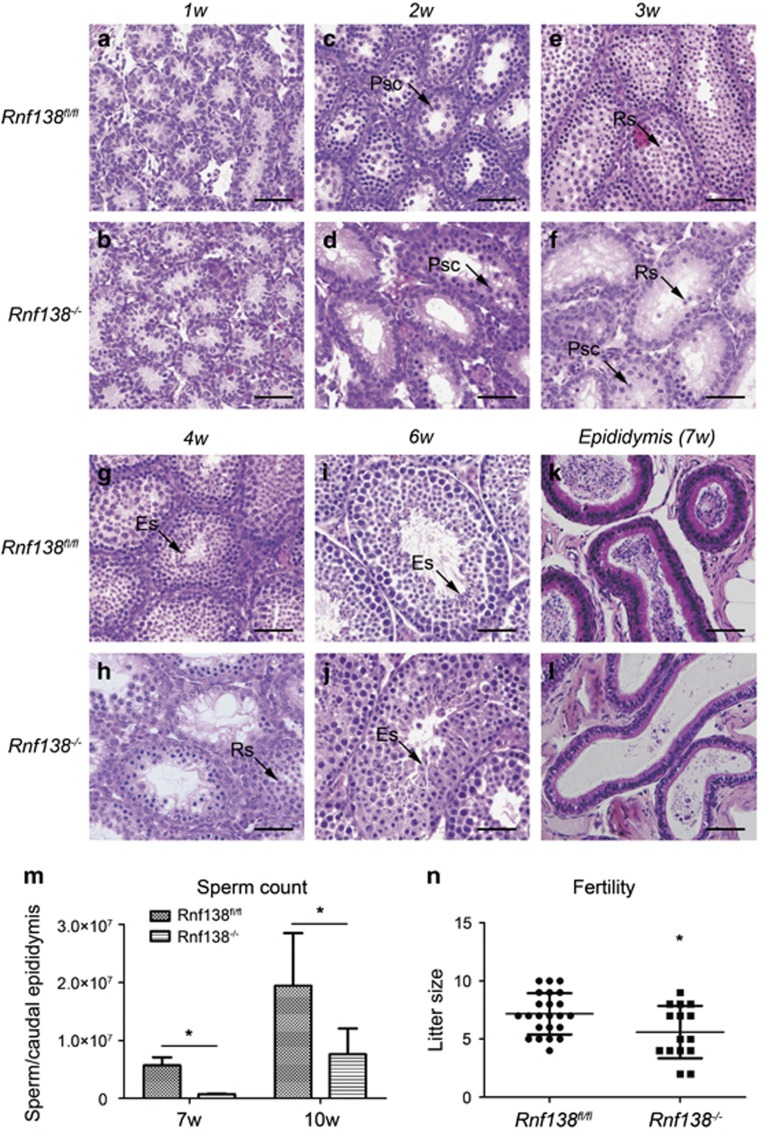
Delayed development of spermatogenesis in the *Rnf138*^*−/−*^ testes. (**a**–**j**) The development of testes from *Rnf138*^*fl/fl*^ and *Rnf138*^*−/−*^ during weeks 1 to 6. The representative seminiferous tubules stained with hematoxylin and eosin (H&E) are shown. Arrows point to primary spermatocytes (Psc), round spermatids (Rs) and elongated spermatids (Es). (**k** and **l**) Representative lumen of epididymis from *Rnf138*^*fl/fl*^ and *Rnf138*^*−/−*^ at 7 weeks of age. (**m**) Sperm counts of caudal epididymis from *Rnf138*^*−/−*^ and control at 7 weeks (*n*=3) and 10 weeks (*n*=5). (**n**) Fertility evaluation of *Rnf138*^*fl/fl*^ and *Rnf138*^*−/−*^. The individual litter sizes (dots) are indicated. Numerical data are shown as mean±S.D. *P<0.05. All scale bars represent 50 *μ*m. w, Weeks

To further characterize the onset and development of the defects in *Rnf138*^*−/−*^ mice, testes from 1- to 6-week-old mice were harvested. Histological analysis revealed obvious structural defects in the *Rnf138*^*−/−*^ mice starting at 2 weeks of age (Figures 4a–d). Spermatocytes markedly emerged in the seminiferous tubules of control testes at 2 weeks of age (Figure 4c). In contrast, 50% of seminiferous tubules only contained a few primary spermatocytes (Figure 4d). At 3 weeks, many round spermatids could be seen in most control seminiferous tubules (Figure 4e). However, only a few round spermatids had developed in *Rnf138*^*−/−*^ testes, although sporadic meiotic cells were seen in some seminiferous tubules (Figure 4f). While round spermatids began to develop into elongated spermatids at 4 weeks in the control testes (Figure 4g), the development of round spermatids was still delayed in *Rnf138*^*−/−*^ tubules with no elongated spermatids at this stage (Figure 4h). In the control, the first wave of spermatogenesis had completed by week 6, and numerous spermatozoa could be found in the control tubules (Figure 4i). However, elongated spermatids just started to appear at week 6 and spermatozoa could be found in only a few of the *Rnf138*^*−/−*^ tubules (Figure 4j).

Some tubules of *Rnf138*^*−/−*^ mice underwent degeneration and displayed a massive loss of spermatocytes with only a single layer of germinal epithelium along the basal membrane from 2 to 4 weeks of age (Figures 4d, f and h). However, surprisingly, the degeneration of tubules disappeared gradually since 5 weeks, and most of the degenerated tubules were recovered in 6-week-old testes (Figure 4j). The knockout of *Rnf138* also delayed the appearance of spermatozoa in the epididymis. After completing the first wave of spermatogenesis at 7 weeks of age, the epididymal lumen was frequently full of spermatozoa in the control group, but mature spermatozoa were rarely seen in the epididymides of *Rnf138*^*−/−*^ mice (Figures 4k and l). The delay until spermatozoa began to emerge in appreciable numbers continued into adulthood in knockout mice. The quantitative analysis of sperm counts showed that the sperm counts of 7 and 10 weeks in *Rnf138*^*−/−*^ mice were significantly lower than in control *Rnf138*^*fl/fl*^ mice (Figure 4m), and two of five 7-week-old *Rnf138*^*−/−*^mice did not have any sperm in the collecting solution of the cauda epididymides. Given the importance of *Rnf138* in regulating juvenile spermatogenesis, we next tested the fertility of adult *Rnf138*^*−/−*^ males. Surprisingly, *Rnf138*^*−/−*^ male mice were fertile, but the size of the litters was significantly smaller than litters fathered by control mice (Figure 4n).

In summary, defects in the seminiferous tubules suggest that the knockout of *Rnf138* delays certain developmental steps in spermatogenesis. The appearance of defects at 2 weeks strongly suggests that *Rnf138* functions at the early development phase of testis, which is the initial development of spermatogonia and spermatocytes.

### *Rnf138* deficiency causes the loss of proliferative spermatogenic cells

**Figure 5:**
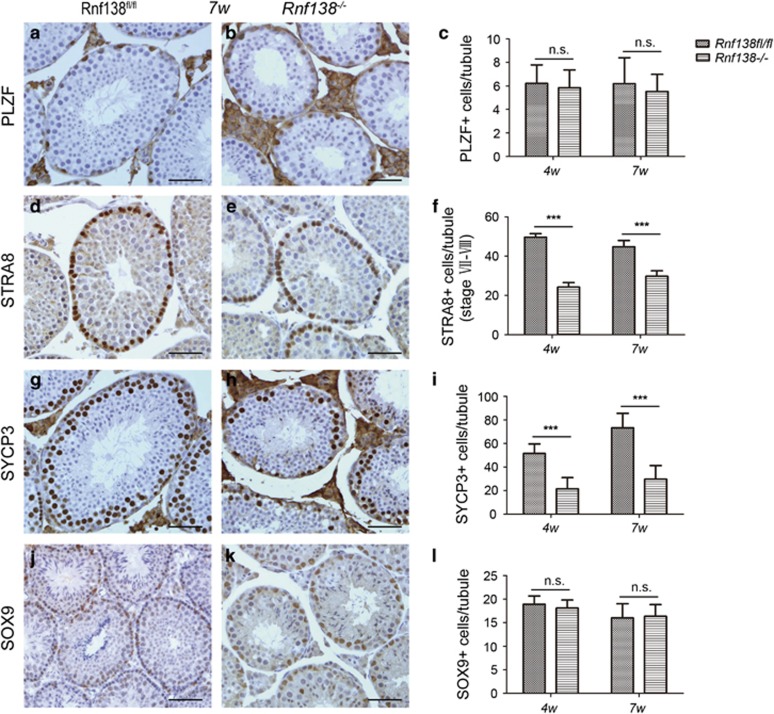
Knockout of *Rnf138* decreases the differentiating spermatogonia. (**a**, **b**, **d**, **e**, **g**, **h**, **j** and **k**) Representative immunostaining of testes at 7 weeks from *Rnf138*^*fl/fl*^ and *Rnf138*^*−/−*^ for PLZF (**a** and **b**), STRA8 (**d** and **e**), SYCP3 (**g** and **h**) and SOX9 (**j** and **k**). The scale bars represent 50 *μ*m. (**c**, **f**, **i** and **l**) Quantitative analyses of PLZF- (**c**), STRA8- (**f**), SYCP3- (**i**) and SOX9- (**l**) positive cells per tubule in the testes at 4 and 7 weeks respectively (*n*=3). Data are presented as mean±S.D. ***P<0.001

To further investigate which types of spermatogenic cells were affected by the knockout of *Rnf138*, the seminiferous tubules were stained with stage-specific markers at 4 and 7 weeks of age. PLZF is widely used as a marker of undifferentiated spermatogonia.^4, 17^ STRA8 is an essential initiator of meiosis in males and is used as a marker for differentiating spermatogonia and preleptotene spermatocytes.^4, 18^ SYCP3 as a axial/lateral element of the synaptonemal complex is a marker for meiotic spermatocytes,^19^ and SOX9 is a key testis-determining gene for specifying the Sertoli cell lineage.^20^ The results showed that the number of PLZF (Figures 5a–c) and SOX9-positive cells (Figures 5j–l) were not affected by *Rnf138*-knockout, suggesting that *Rnf138* does not have a role in the maintenance of Sertoli cells and undifferentiated spermatogonia. In contrast, the numbers of STRA8-positive cells (Figures 5d–f) as well as SYCP3-positive meiotic spermatocytes (Figures 5g–i) were significantly decreased in the *Rnf138*^*−*/*−*^ testes at both ages. This implied that *Rnf138* may promote the development of differentiating spermatogonia cells, which correlates well with the appearance of structural defects at 2 weeks of age and the testicular localization of RNF138.

**Figure 6:**
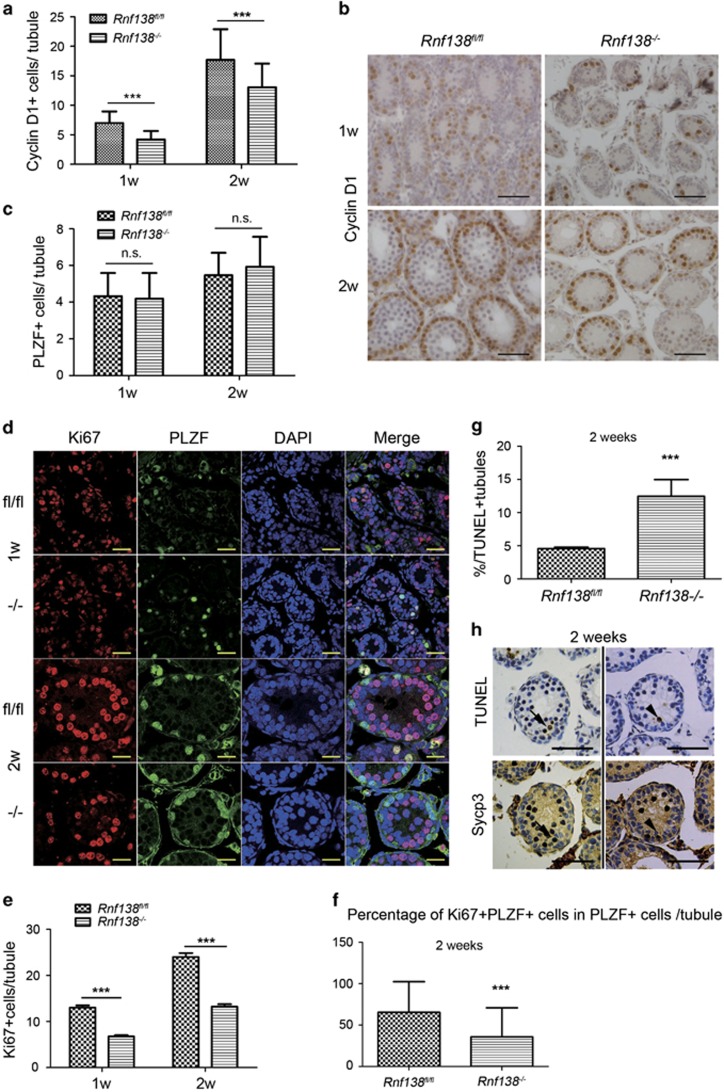
Knockout of *Rnf138* promotes apoptosis and causes loss of proliferative spermatogonia. (**a**) Quantitative analysis of cyclin D1-positive cells per tubule in the testes of *Rnf138*^*−/−*^ and control mice (*n*=3) at 1 and 2 weeks. (**b**) Representative immunostaining of cyclin D1 on testes sections at 1 and 2 weeks of age. The scale bar represents 50 *μ*m (**c**) Quantitative analysis of PLZF-positive cells per tubule at 1 and 2 weeks testes from *Rnf138*^*−/−*^ and control (*n*=3). (**d**) Immunostaining for Ki67 (red) and PLZF (green), with 4',6-diamidino-2-phenylindole (DAPI) counterstain (blue), on control and *Rnf138*^*−/−*^ testes at 1 and 2 weeks. Scale bars=30 *μ*m. (**e**) Quantitative analysis of Ki67-positive cells per tubule in the testes of *Rnf138*^*−/−*^ and control mice (*n*=4) at 1 and 2 weeks. (**f**) The proportion analysis of Ki67+PLZF+ cell within the PLZF+ population in each tubule in the testes of *Rnf138*^*fl/fl*^ and *Rnf138*^*−/−*^ mice (*n*=4) at 2 weeks. (**g**) Quantification of tubules with TUNEL-positive cells showed the increase of tubules with apoptotic testicular cells in *Rnf138*^*−/−*^ at weeks 2 (*n*=4). (**h**) TUNEL and immunostaining for SYCP3 on serial sections in the testes of *Rnf138*^*−/−*^ mice at 2 weeks. Apoptotic spermatogonia (arrowhead) and spermatocyte (arrow) were detected. Scale bars=50 *μ*m. All values are mean±S.D. ***P<0.001; NS, no significance

We assessed the differentiation of spermatogonia at weeks 1 and 2. STRA8-positive cells first appeared at 2 weeks in both *Rnf138*^*−/−*^ and control tubules, and the number of STRA8-positive cells per tubule was still less in *Rnf138*^*−/−*^ than in control (data not shown). As spermatogenic cells were much less in some *Rnf138*^*−/−*^ tubules, it was hypothesized that *Rnf138* may have a role in the proliferating spermatogonia. We immunostained testes sections of cyclin D1, a marker for proliferating spermatogonia, which is present at highest levels at the G1 and G2 phases of the cell cycle.^21^ Intriguingly, the number of cyclin D1-positive cells significantly decreased in *Rnf138*^*−/−*^ testes compared with control testes at both 1 and 2 weeks of age (Figures 6a and b). To confirm if this was caused by the difference in undifferentiated spermatogonia, PLZF-positive cells were examined at 1 and 2 weeks. However, similar to other ages, the population of PLZF-positive cells was not significantly affected by the knockout (Figure 6c). This suggests that knockout of *Rnf138* promotes the great loss of proliferative spermatogonia. To further confirm this hypothesis, we costained the PLZF and Ki67, which is expressed at all dividing cell-cycle stages. Similar to cyclin D1, Ki67 was present at very low levels in *Rnf138*^*−/−*^, and the number of Ki67-positive cells per tubule was significantly lower than that of control at both 1 and 2 weeks (Figures 6d and e). Then, we analyzed the percentage of Ki67+PLZF+ cells within the PLZF+ population in each tubule at 2 weeks. The percentage was significantly higher in *Rnf138*^*fl/fl*^ than in the *Rnf138*^*−/−*^(Figure 6f), which suggests a delay in spermatogonia differentiation.

Meanwhile, the apoptotic index was significantly higher in *Rnf138*^*−/−*^ than in control at 2 weeks (Figure 6g). To understand the type of apoptotic cells in *Rnf138*^*−/−*^, we then carried out a TUNEL assay and immunostaining for SYCP3 (a marker of meiotic spermatocyte), respectively, on serial sections from *Rnf138*^*−/−*^ at 2 weeks. The majority of TUNEL-positive cells were spermatogonia cells where the SYCP3 staining was negative, and a few of TUNEL-positive cells were spermatocytes (Figure 6h). These results indicates that the knockout of *Rnf138* promotes the apoptosis of proliferative spermatogenic cells, especially the differentiating spermatogonia.

### RNF138 is not essential for meiotic recombination

RNF138 was reported to be a key factor in the regulation of HR DNA repair, and then we wondered if *Rnf138* had a role in meiotic HR in spermatocytes I. SPO11 cleaves the chromosomal DNA to generate double-strand break (DSB), which initiates the meiotic recombination.^22^ Then, DNA repair events during prophase I were detected in spermatocytes from *Rnf138*^*−*/*−*^ at 4 weeks. Chromosome spreads were stained with antibodies against specific markers of DSB induction and repair. The dynamics of DSB repair were similar in spermatocytes from *Rnf138*^*fl/fl*^ and *Rnf138*^*−/−*^. The appearance of phosphorylated form of histone H2AX, known as γH2AX, indicates the sites of DSB in leptonema. As DSBs were processed by the appropriate repair machineries, γH2AX signals declined, marking the progression from leptonema through zygonema. By pachynema, residual γH2AX was located at the sex chromosome only, and diminished through diplonema (Supplementary Figure 3sA). RAD51 as an early recombination protein facilitates early events of strand invasion following DSB induction and marks all DSB events that are destined to be crossovers (COs) or non-COs. A large number of RAD51 foci at leptonema and zygonema were detected at both the groups and were almost entirely absent at pachynema and diplotene (Supplementary Figure 3sB). Synapsis normally occurred at late zygonema and completed by pachynema in spermatocytes from *Rnf138*^*−/−*^ (Supplementary Figure 3sC). To further explore the HR process in meiosis, chromosome spreads were stained for the CO marker MLH1, which can be used as a metric for the majority of CO events.^23^ The result showed that the MLH1 foci could be normally detected on the DSB repair sites in pachytene spermatocyte from *Rnf138*^*−/−*^ (Supplementary Figure 3sD). Collectively, all these results indicates that *Rnf138* may not be essential for the male meiotic recombination.

### Effects of *Rnf138* deficiency on gene expression

**Figure 7:**
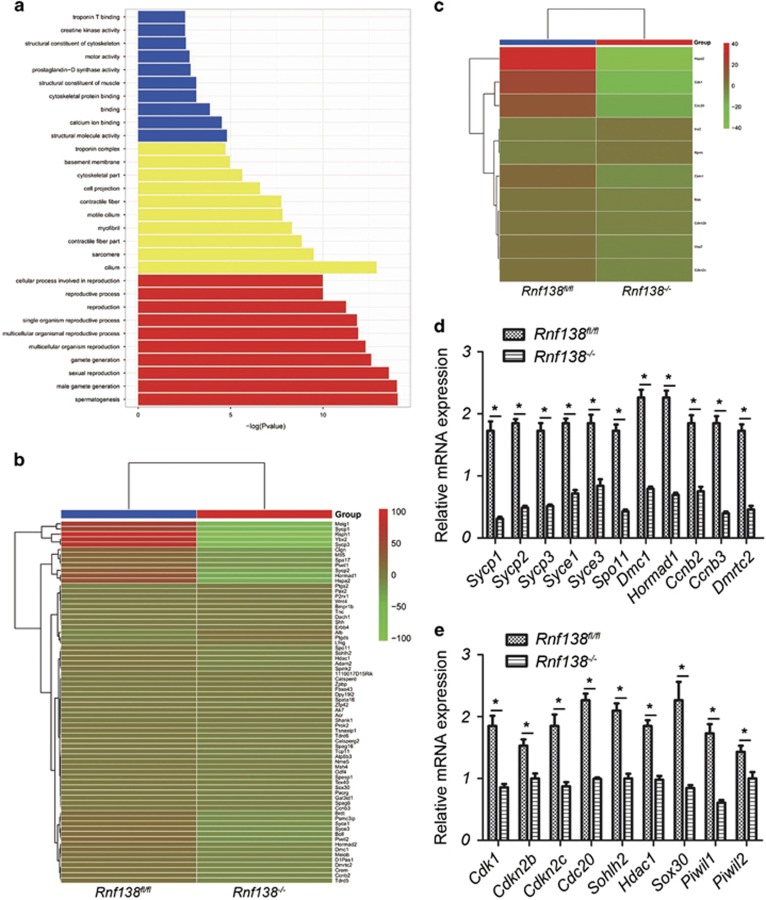
Gene expression analysis. (**a**) GO categories for the genes with fold change ⩾2. (**b** and **c**) Heat map of an individual cluster of significantly changed genes involved in spermatogenesis (**b**) and cell cycle (**c**). Each column represented a genotype shown at the bottom, and each row corresponded to the relative expression prolfile of each gene. Results were expressed as Fragments Per Kilobase of transcript per Million (FPKM) (*n*=3). (**d** and **e**) Quantitative real-time PCR analysis on 2-week testis for genes differentially expressed between *Rnf138*^*fl/fl*^ and *Rnf138*^*−/−*^. Each gene is indicated at the bottom of the respective column. Experiments were repeated three independent times. Data were presented as mean±S.E.M. *P<0.05

We next performed RNA-seq analysis on 2-week-old testes to identify molecular disturbances that might precede the histopathological characteristics of *Rnf138*^*−/−*^ mice. Three independent RNA-seq experiments were performed on three pairs of *Rnf138*^*fl/fl*^ and *Rnf138*^*−/−*^ testes. We focused on the mRNA expression of genes with known or suspected functions in spermatogenesis. Among all genes with altered expression profiles in *Rnf138*^*−/−*^ testes, 628 genes had a statistically significant fold change ⩾2 compared with *Rnf138*^*fl/fl*^ testes (Supplementary s1). Of these, 406 genes were downregulated and 222 genes were upregulated. Functional analysis of the 628 genes determined that the gene ontology (GO) enrichment of altered gene expression was mainly associated with spermatogenesis (Figure 7a and Supplementary s2). Consistent with our phenotype, loss of *Rnf138* also affected genes responsible for spermatogenesis, differentiation and cell cycle (Figures 7b and c and Supplementary s3).

RNA-seq findings were confirmed by qRT-PCR on 2-week-old testes for a group of genes known to be involved in spermatogenesis. Spermatogenic cells with impeded entrance into meiotic phase should not engage in meiotic recombination, and the expression of *Spo11* and *Dmc1* is markedly reduced in *Rnf138*^*−/−*^.^24^ Components of the synaptonemal complex, *Sycp1*, *Syce1*, *Syce2*, *Syce3*, *Tex12*, *Sycp2*, *Sycp3* and *Hormad1*, were significantly downregulated in *Rnf138*-knockout animals, indicating that at 2 weeks of age, *Rnf138*-deficient testes performed low levels of DSBs and synaptonemal complex formation (Figure 7d). In addition, a number of genes involved in meiosis, for example, *Ccnb2*, *Ccnb3* and *Dmrtc2*, were also downregulated in *Rnf138*-knockout testes (Figure 7d). The altered gene expression explains the low proportion of meiosis, which corresponds with the phenotype of 2-week-old testes in which a small number of primary spermatocytes appeared in the tubules in *Rnf138*^*−/−*^ testes. In addition to the aforementioned genes, the important cell-cycle regulators *Cdk1*, *Cdkn2b* and *Cdc20* were significantly downregulated in *Rnf138*^*−/−*^ testes (Figure 7e). The downregulation of *Cdkn2c*, which is important for the transition from mitosis to meiosis, was further confirmed by qRT-PCR (Figure 7e). *Sohlh2*, *Piwil1* and *Piwil2* are genes involved in spermatogonia differentiation, and they were all downregulated in the knockout mice (Figure 7e). *Sox30*, which was also downregulated, belongs to the SOX family of transcription factors, but its function in testes remains unknown (Figure 7e). The epigenetic modification gene *Hdac1* was significantly downregulated in *Rnf138*^*−/−*^ (Figure 7e). The aberrant expression of these genes may have been caused by the loss of proliferative spermatogonia and delayed entrance into meiosis in the knockout mouse. Further study is needed to better define the role of *Rnf138* between apoptosis and proliferation.

## Discussion

E3 ligases have been reported to have an important role in testis development and spermatogenesis. Even though the E3 ligase RNF138 is highly expressed in testis, its function in spermatogenesis remains poorly understood. In this study, we characterized the detailed localization and function of RNF138 in mouse testes, to the best of our knowledge for the first time, which extended the knowledge in this unknown field.

The expression of *Stra8* is greatly upregulated in stage VI–VIII tubules where differentiating spermatogonia and preleptotene spermatocytes emerge, which are exposed to the highest levels of retinol acid.^4, 18, 25^ STRA8-positive cells in stage VII–VIII tubules display the preleptotene and differentiating type A spermatogonia phenotype.^18^ The decreased population of STRA8-positive cells in stage VII–VIII tubules suggested the loss of differentiating spermatogonia and preleptotene spermatocytes. Since sperm can be successfully produced and *Rnf138*^*−/−*^ males are fertile, it is confirmed that the defects display before the initiation of prophase I, which proceeds normally. The differentiating spermatogonia should be the cell type mainly affected by the knockout.

Undifferentiated spermatogonial progenitors are ultimately committed to differentiation. In a series of cell divisions, undifferentiated A-type spermatogonia differentiate into B-type spermatogonia, which further proliferate and differentiate into meiotic spermatocytes.^3^ Cyclin D1 is expressed only in proliferating spermatogonia.^26^ Meanwhile, the expression of PLZF is restricted to undifferentiated spermatogonia that are negative for cyclin D1 and thus largely quiescent.^27^ The knockout of *Rnf138* significantly reduced the number of cyclin D1-positive spermatogonia, indicating that *Rnf138* deficiency disrupts the proliferation of differentiating spermatogonia. The result of Ki67 staining confirmed this conclusion. The structural defects at 2 weeks of age also support this conclusion.

The differentiation step from A_al_ to A_1_ spermatogonia brings about a marked change of proliferation pattern. A_s_, A_pr_ and A_al_ spermatogonia proliferate at random during a particular period, while the proliferation of A1–B spermatogonia is highly synchronized, and when they are unable to divide at the appropriate time, they enter apoptosis.^3^ The lower proportion of Ki67+PLZF+ spermatogonia within the whole PLZF+ population indicates the aberrant proliferation in *Rnf138*^*−/−*^. However, the proliferation of Ki67-positive cells did not affect the population of undifferentiated spermatogonia (the PLZF-positive spermatogonia), which indicated that the proliferating spermatogonia underwent apoptosis during the differentiation process. The impaired proliferation of spermatogonia may have contributed to the increased apoptosis of spermatogenic cells, especially the differentiating spermatogonia, which accounts for most of the decreased cyclin D1-positive or Ki67-positive cells. It is therefore likely that the knockout of *Rnf138* impaired the proliferation of differentiating spermatogonia, and the reduction in differentiating spermatogonia accounts for the loss of preleptotene spermatocytes and low levels of meiosis.

The efficiency of cell proliferation and complex differentiation in spermatogonia relies on the coordinated control of gene expression. *Cdk1* has been proposed to be a master regulator of the mammalian cell cycle because of its complex association with several major mitotic cyclins,^28^ and *Cdkn2b* is a cell growth regulator that controls cell-cycle G1 progression with unknown functions in spermatogenesis. *Cdc20*, the activating subunit of the anaphase-promoting complex, is essential for cell-cycle progression.^29^ The differentiation of spermatogonia is a process of cell proliferation, which may have been inhibited by the abnormal downregulation of these genes. The altered transcription of these essential cell-cycle genes in *Rnf138*^*−/−*^ suggests that *Rnf138* may have a role in regulating the proliferation of spermatogenic cells. The combined loss of *Cdkn2c* and *P19*^*Ink4d*^ is associated with the delayed exit of spermatogonia from the mitotic cell cycle, leading to the retarded appearance of meiotic cells that do not properly differentiate and instead undergo apoptosis at an increased frequency.^30^ The detected apoptotic spermatocytes proved this. The downregulation of proliferation-associated genes and the meiosis genes such as *Spo11*, *Dmc1*, *Sycp1, Sycp2* and *Sycp3* proved that the insufficient proliferation of spermatogonia delayed the development of spermatogenic cells, resulting in the loss of spermatogonia and spermatocytes in *Rnf138*^*−/−*^ testes.

In conclusion, our data demonstrate that RNF138, which is highly expressed in mouse testis, locates mainly in spermatogonia and spermatocytes. Knockout of *Rnf138* delays testis development, at least in part, by promoting the apoptosis of proliferative spermatogonia. *Rnf138* may have regulated the proliferation of spermatogonia and prevented the apoptosis of spermatogenic cells. Our study provided an important function indication of *Rnf138* in testis development, and its detail function in regulating the proliferation of spermatogonia may need further study.

## Materials and Methods

### RNF138 antibody

Rabbits were immunized with a C-terminal mouse RNF138 fragment containing amino-acid residues 60–245 and N-terminal His and T7 tags that was first purified from *Escherichia coli*. Serum antibodies were purified on SulfoLink Coupling Resin affinity columns (Thermo Fisher Scientific, Waltham, MA, USA). Antibodies were produced and purified by ABclonal (Wuhan, China).

### Targeting vector and mice

All animal experiments were approved by the Animal Care and Use Committee of the Institute of Basic Medical Sciences, Chinese Academy of Medical Sciences, and were performed following the Guiding principles for the care and use of research animals. Mice in which the second exon of *Rnf138* is flanked by *lox*P sites (*Rnf138*^*fl/fl*^) and transgenic *Vasa*-*Cre* mice, expressing Cre recombinase under the control of the germline-specific promoter *Vasa*, were purchased from the Model Animal Research Center of the National Resource Center for Mutant Mice (Nanjing, China). The targeting vector designed to replace the floxed *Rnf138* exon 2 contained a neomycin cassette flanked by *FLP* recombinase target sequences, the 5′-homologous arm and the 3′-homologous arm (Supplementary Figure 1sB). The vector was electroporated into mouse embryonic stem (ES) cells, and the targeted ES cells were injected into blastocysts from C57BL/6 mice. Genomic DNA was isolated from tail snips and pups were genotyped for *Rnf138* deletion by PCR amplification using the following primers: common *Rnf138* forward primer (primer 1), 5′-TATAGTTCTGGCTCTCTGAA-3′ reverse primer for WT and floxed *Rnf138* (primer 2), 5′-ATTTGTGACAGGTTAATTAC-3′ reverse primer for exon 2 deletion (primer 3), 5′-CTGACTTGGGTAAATGCTCAAT-3′ (Supplementary Figure 1sB). Primers for *Vasa*-*Cre* genotyping: forward primer, 5′-CACGTGCAGCCGTTTAAGCCGCGT-3′ reverse primer, 5′-TTCCCATTCTAAACAACACCCTGAA-3′. All mice used in the experiments were housed in the Center for Experimental Animal Research at the Chinese Academy of Medical Sciences.

### Immunoblot analysis

Cells and tissues were harvested and lysed in protein extraction buffer containing 50 mM Tris-HCl (pH 6.8), 2% SDS and 10% glycerol. Total protein was quantified by BCA Protein Assay (Pierce, Waltham, MA, USA), and samples were subjected to immunoblotting as described previously.^31^

### Histology, immunohistochemistry and immunofluorescence

Testes and epididymides were harvested from mice and fixed at 4 °C in Bouin's solution (saturated picric acid: formaldehyde: glacial acetic acid 15:5:1) or 4% paraformaldehyde. Fixed tissues were dehydrated, paraffin-embedded and sectioned. Sections were deparaffinized and rehydrated before staining.

Sections for immunohistochemistry were first boiled in antigen retrieval buffer (0.001M EDTA, 0.01M Tris-Base (pH 9.0)) for 10 min and then incubated in 3% H_2_O_2_ for 10 min at room temperature to block endogenous peroxidase activity. Slides were then washed with PBS (0.14M NaCl, 0.0027M KCl, 0.01M Na_2_HPO_4_ and 0.002M KH_2_PO_4_, pH 7.4) and blocked in 10% normal goat or rabbit serum in PBS for 30 min at room temperature. Sections were incubated in primary antibody diluted in block solution at 4 °C overnight.

All remaining wash, incubation and detection steps were performed using the Polink-2 HRP Plus Polymer Detection System and DAB Kit (ZSGB-BIO, Beijing, China) according to the manufacturer’s instructions. The following antibodies were used in this study: rabbit polyclonal anti-RNF138 (ABclonal, custom antibody); rabbit polyclonal anti-STRA8 (no. ab49602; Abcam, Cambridge, UK); mouse monoclonal anti-PLZF (no. sc-28319; Santa Cruz, Dallas, TX, USA); mouse monoclonal anti-SYCP3 (no. ab97672; Abcam); rabbit polyclonal anti-SOX9 (no. AB5535; Millipore, Darmstadt, Germany); rabbit monoclonal anti-cyclin D1 (no. ab134175; Abcam).

Immunofluorescence was performed on PFA-fixed paraffin-embedded sections. The procedure before the incubation of primary antibody was similar to immunohistochemistry. After the incubation of primary antibodies, sections were washed with PBS and blocked in 10% normal goat serum in PBS for 60 min at room temperature. Then, the sections were incubated with the secondary anibody diluted in block solution at room temperature in a darkroom. Sections were then washed with PBS. Mounting medium with DAPI (ZSGB-BIO, ZLI-9557) was added and slides were covered with coverslips. Primary and secondary antibody used were as follows: rabbit monoclonal anti-Ki67 (no. ab16667; Abcam); mouse monoclonal anti-PLZF (no.sc-28319; Santa Cruz); secondary antibody (A21202 and A21207; Molecular Probes, Eugene, OR, USA).

TUNEL assay was carried out using the *In Situ* Cell Death Detection Kit, POD (no. 11684817910; Roche Applied Science, Indianapolis, IN, USA), according to the manufacturer’s instructions.

### Chromosome spreads and immunofluorescence

Glass slides were pretreated in methanol solution containing 0.4‰ HCl. Testis was dissected and the capsule was removed in PBS. Seminiferous tubules were gently separated with forceps and incubated in a Petri dish for 25 min containing 5 ml of HEB (30 mM Tris (pH 8.2), 50 mM sucrose, 17 mM citric acid, 5 mM EDTA, 2.5 mM DTT, 1 mM PMSF). Then, the cells were released from the tubules by gently pipetting in and out in 0.1M sucrose. A total of 20–30 *μ*l of cells was added to the glasses covered by 1% PFA. The cells were allowed to adhere by resting the glass in a humidity chamber overnight. Air dry the glasses in room temperature, then immersed the glasses in 0.4‰ Photo-Flo200 (Kodak, Rochester, NY, USA) for 4 min and air dried. The slides were then incubated with 1 × ADB (1% normal donkey serum, 0.3% BSA and 0.05‰ Triton X-100 in TBS (0.15M NaCl, 0.5M Tris-HCl, pH 7.6)) blocking solution for 30 min in a humidity chamber. Primary antibodes were then diluted in ADB solution and incubated at 37 °C overnight under the same condition. In case of dry out, slides can be sealed by rubber cement. After three times 10 min washes in PBS, slides were blocked by 1 × ADB for another night at 4 °C. Then, slides were washed in precooled TBST for another three 10 min, and incubated with secondary antibodies for 1–2 h at 37 °C. Slides were then washed three times in TBST, and allowed to dry out. Vectashield Mounting Medium with DAPI (no. H-1000; Vector Laboratories, Burlingame, CA, USA) was added and slides were covered with coverslips. Slides were viewed with an Olympus confocal microscope (Tokyo, Japan). Commercial primary antibodies were used as follows: anti-*γ*H2AX (no.05-636; Millipore), anti-Rad51 (sc-8349; Santa Cruz), anti-SYCP3 (ab97672 and ab15093; Abcam), anti-MLH1 (551092; BD Pharmingen, San Diego, CA, USA), anti-SYCP1 (NBP300-229; Novus, Littleton, CO, USA).

### Epididymal sperm count

To determine the sperm count, the right cauda epididymis was collected using surgical scissors, placed in 1 ml PBS and minced to allow the sperm to swim out for 30 min at 37 °C. Sperm samples were diluted with Trypan blue staining solution (0.25% Trypan blue, 5% NaHCO_3_, 0.35% formalin), and the total number was determined using a hemocytometer.

### RNA-seq, qRT-PCR and RT-PCR

Reads generated by RNA-seq ranged from 12-24G per library. Qualified sequencing reads were mapped to the mouse genome GRCm38 using Bowtie2 and TopHat2. Aligned RNA-seq reads were assembled into transcripts using the Cufflinks program according to Gencode v.M8 (http://www.gencodegenes.org/), and transcript abundance was estimated in fragments per kilobase of exon per million fragments mapped (FPKM). Hypergeometric distribution was used to determine GO enrichment.

Total testis RNA was reverse transcribed using the TransScript First-Strand cDNA Synthesis Kit (TransGen, Beijing, China) according to the manufacturer’s instructions. Quantitative Real-time PCR (2 × SYBR Green Fast qPCR Master Mix; Biotool, Houston, TX, USA) and RT-PCR were performed using the primers listed in Supplementary Table 1s. Gene expression was normalized to actin.

### Fertility evaluation

Seven-week-old adult male control or knockout mice were bred with wild-type fertile adult females at a male : female sex ratio of 1/2 for up to 1 month and the resulting litter sizes were analyzed as a measure of fertility.

### Statistical analysis

All experiments were independently repeated at least three times. Data were analyzed by two-way unpaired Student’s *t*-test. All statistical analyses were performed using the GraphPad Prism Software (La Jolla, CA, USA). Differences were considered statistically significant at *P*<0.05.
