# Supplementary figures and images for: Rnf138 deficiency promotes apoptosis of spermatogonia in juvenile male mice
Source: Cell Death Dis. 2017 May 18;8(5):e2795–. doi: 10.1038/cddis.2017.110 (PMC5520686; doi:10.1038/cddis.2017.110)

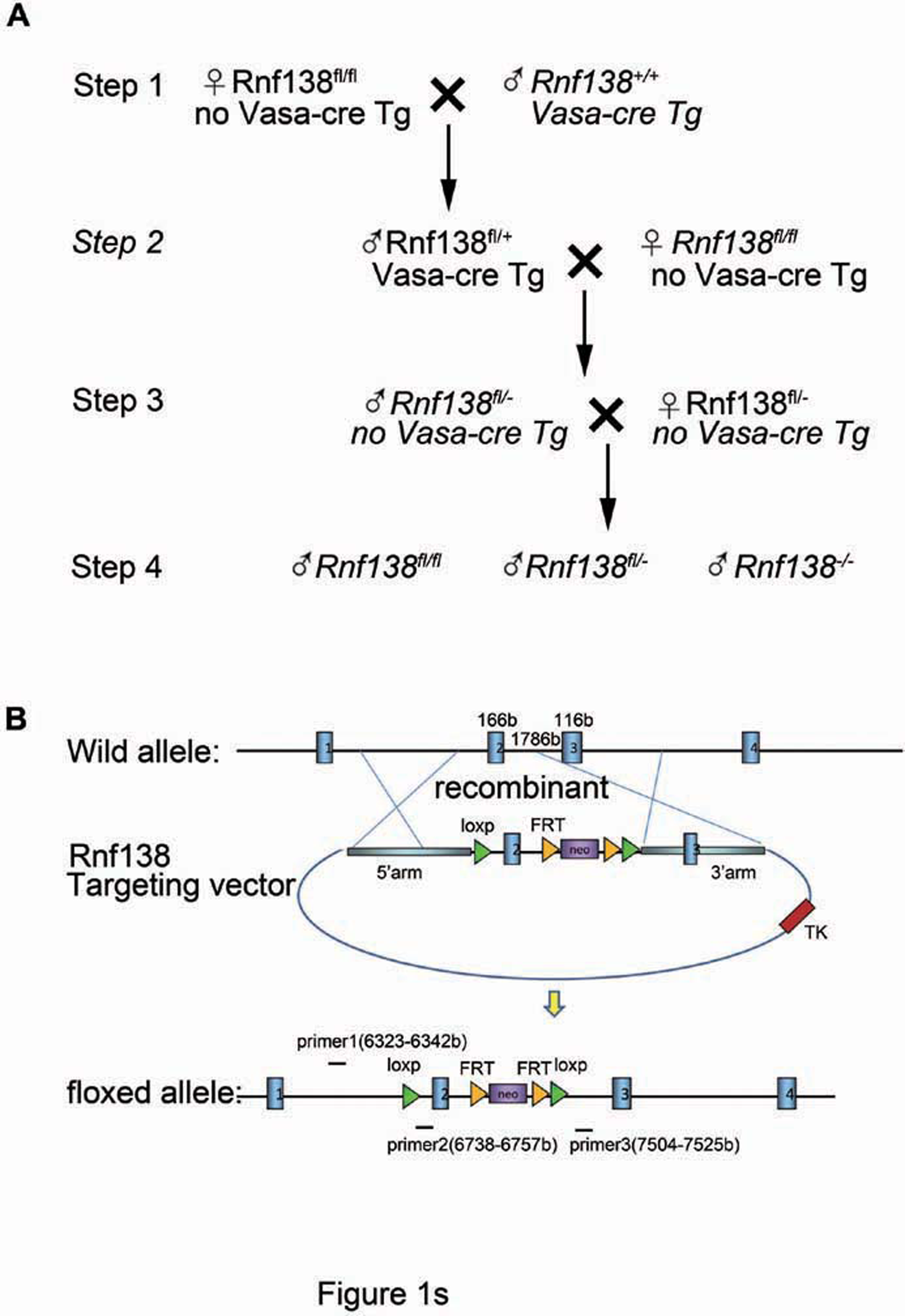

Supplement: Supplementary Figure S1 [file cddis2017110x1.tif]

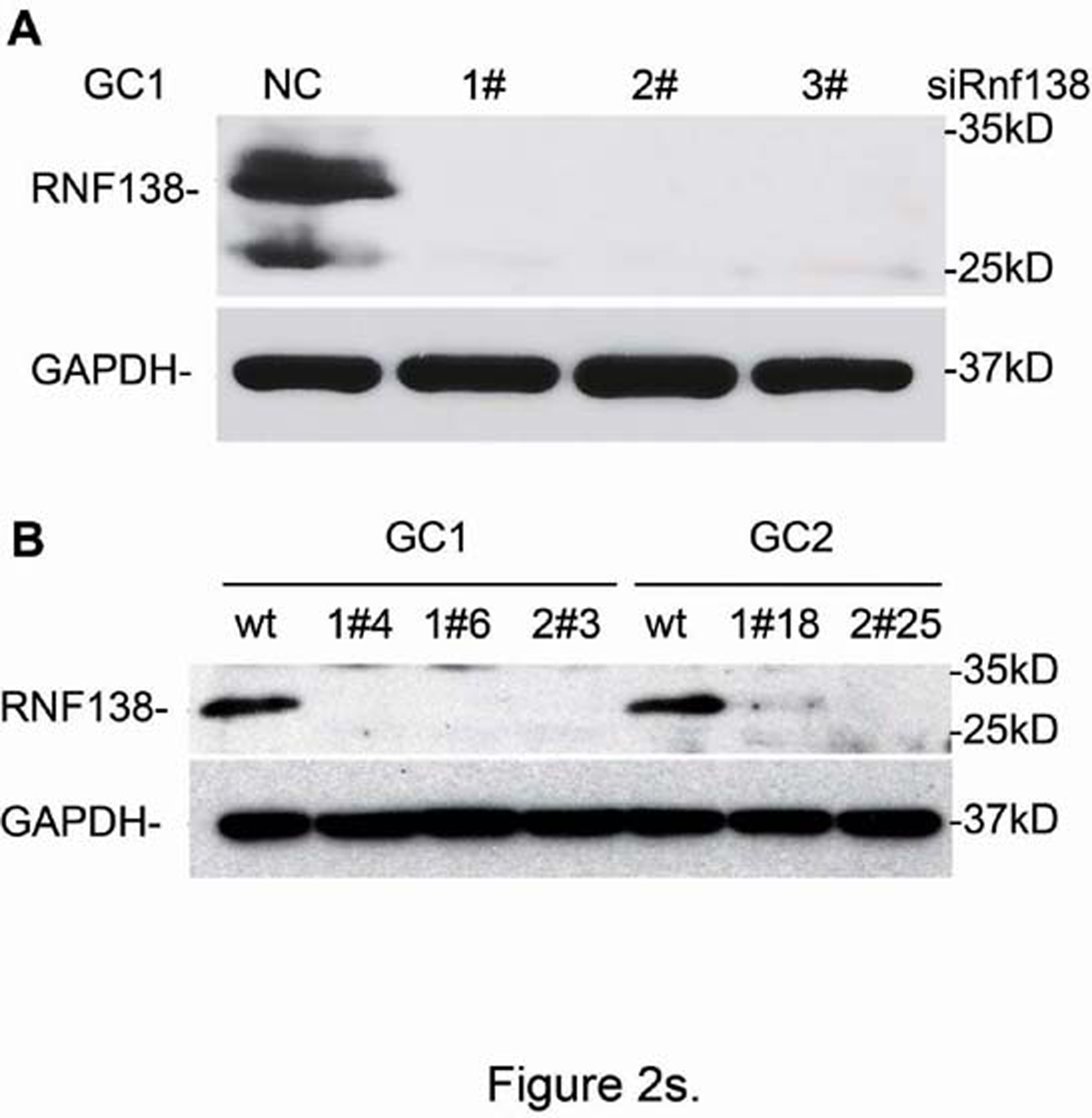

Supplement: Supplementary Figure S2 [file cddis2017110x2.tif]

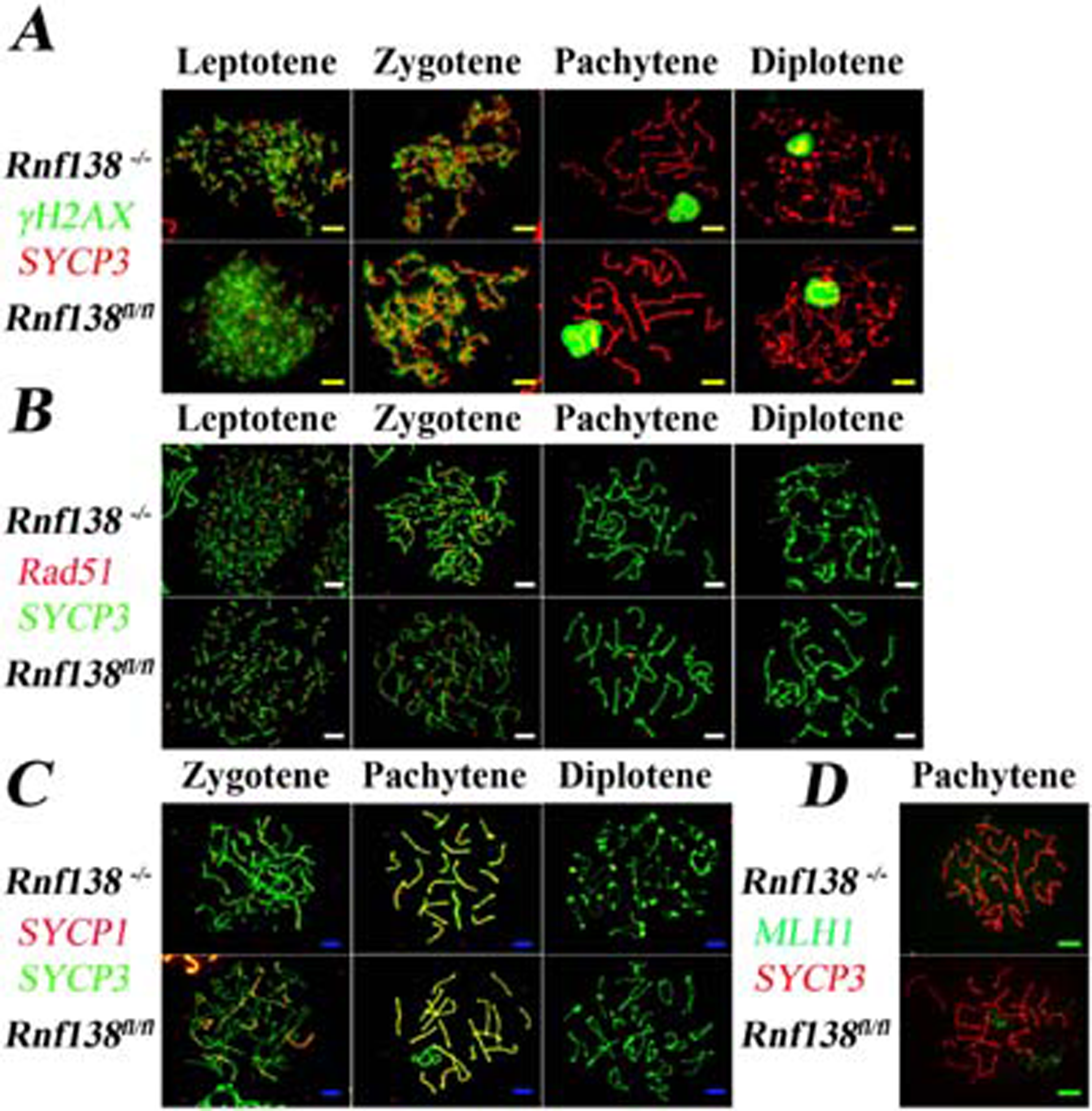

Supplement: Supplementary Figure S3 [file cddis2017110x3.tif]
