# Supplementary material for: Rnf138 deficiency promotes apoptosis of spermatogonia in juvenile male mice
Source: Cell Death Dis. 2017 May 18;8(5):e2795–. doi: 10.1038/cddis.2017.110 (PMC5520686; doi:10.1038/cddis.2017.110)
Supplement: Supplementary Table S1 [file cddis2017110x4.docx]

| Targeted gene | Sequence of primer1 | Sequence of primer1 | Application |
| --- | --- | --- | --- |
| Ccnb2 | GCCAAGAGCCATGTGACTATC | CAGAGCTGGTACTTTGGTGTTC | qRT-PCR |
| Ccnb3 | AGAGAAACCTGTGATTCAGGAGA | GAAATGCGGCTTTTTGACTGG | qRT-PCR |
| Cdk1 | AGAAGGTACTTACGGTGTGGT | GAGAGATTTCCCGAATTGCAGT | qRT-PCR |
| Cdkn2b | CCCTGCCACCCTTACCAGA | CAGATACCTCGCAATGTCACG | qRT-PCR |
| Cdkn2c | CCTTGGGGGAACGAGTTGG | AAATTGGGATTAGCACCTCTGAG | qRT-PCR |
| Cdc20 | TTCGTGTTCGAGAGCGATTTG | ACCTTGGAACTAGATTTGCCAG | qRT-PCR |
| Hdac1 | AGTCTGTTACTACTACGACGGG | TGAGCAGCAAATTGTGAGTCAT | qRT-PCR |
| Sox30 | TCACACCAACTGACCCAGAA | TCGGTTCTCCTTTCATCACC | qRT-PCR |
| Sycp1 | ATGGAGAAGCAAAAGCCCTTC | TTTCTGCTTCAGTTCAGATTC | qRT-PCR |
| Sycp2 | GACACTGAAACCGAATGTGGA | TGTGGGTCTTGGTTGTCCTTT | qRT-PCR |
| Sycp3 | GAAATCTGGGAAGCCACCTT | GCTCCAAATTTTTCCAGCAT | qRT-PCR |
| Syce1 | GCATGTTGCAGGAGTGTAAAGA | GCTGCTGTCCAAAACACACATC | qRT-PCR |
| Syce3 | CCTGGATGGCCTATGATATGGT | GGAAGGCGTCTTCTAGCCG | qRT-PCR |
| Spo11 | CGGCTCCTGGACGACAACT | ACGCCCTTTCCCGTAACC | qRT-PCR |
| Dmc1 | TTCGTACTGGAAAAACTCAGCTGTCTC | CTTGGCTGCGACATAATCAAGTAGCTCC | qRT-PCR |
| Hormad1 | GAGGACAGCTTCCCTGAGTG | AAAGCATCATAGCATCCAAG | qRT-PCR |
| Piwil1 | TAAGTGAAGTATCCAGGTG | GTCCCAATTATCAAAGAAGC | qRT-PCR |
| Piwil2 | AGAGGTTGGCGAGGAATAAGG | CATTATGGTCAAGTATCTGTT | qRT-PCR |
| Dmrtc2 | CCCTTCTGGAAAAGAGAACATAGC | CTTGGCACAGCAAGCGGCATACCA | qRT-PCR |
| Sohlh2 | TCTCAGCCACATCACAGAGG | GGGGACGCGAGTCTTATACA | qRT-PCR |
| Actin | CCCAACTTGATGTATGAAGG | TTGTGTAAGGTAAGGTGTGC | qRT-PCR |
| Rnf138 | CCACGTCCTACACGGAAGATG | CCACTTTCTCTCATTGCAGTCA | qRT-PCR |
| Rnf138 | ATGTCCGAGGAACTTTCGG | CATGTTTACTTGAAAAGACTCTTCCAC | RT-PCR |
| Gapdh | ATGGTGAAGGTCGGTGTCAAC | TTACTCCTTGGAGGCCATG | RT-PCR |

Primers used in the study
