# Supplementary material for: Rnf138 deficiency promotes apoptosis of spermatogonia in juvenile male mice
Source: Cell Death Dis. 2017 May 18;8(5):e2795–. doi: 10.1038/cddis.2017.110 (PMC5520686; doi:10.1038/cddis.2017.110)
Supplement: Supplementary Figure Legends [file cddis2017110x9.docx]

**Supplemental data legends**

**Figure 1s. Strategy for the generation of *Rnf138^-/-^*.** (A) Mating strategy for the generation of *Rnf138^-/-^*. Vasa-cre Tg indicates mouse carrying the Vasa-cre transgene. (B) Targeting strategy for a *loxP*-floxed *Rnf138* allele in ES cells. Based on homologous recombination, the exon 2 of *Rnf138 was* flanked *by two lox*P sites, and a neomycin cassette flanked by two *FRT* was introduced between exon 2 and the *lox*P at 3-terminal. Primer1, common *Rnf138* forward primer; Primer2, reverse primer for WT and floxed *Rnf138*; Primer3, reverse primer for exon 2 deletion.

**Figure 2s. Specificity analysis of RNF138 antibody in GC1 and GC2 cells by knockdown and knockout.** (A) Western blot analysis of knock-down of RNF*138* in GC1 cells. (B) Western blot analysis of knockout of *Rnf138* by *CRISPR-Cas9* in GC1 and GC2 cells. 1#4, 1#6,2#3, 1#18 and 2#25 represented different cell clones ablated *Rnf138*. The immunoblots are representative of three replicates.

**Figure 3s. Normal prophase Ⅰ progression in *Rnf138^fl/fl^* and *Rnf138^-/-^* males.** (A) Double strand break processing was monitored by stainingγH2AX (green) and SYCP3 (red) to highlight the location and frequency of DSBs. (B) Rad51 (red) was stained to highlight initial events of DSB repairing. (C) SYCP1 (red) and SYCP3 (green) were stained to detect the formation of chromosome synapsis in *Rnf138^fl/fl^* and *Rnf138^-/-^* during prophaseⅠ. Synapsis is observed by the co-localization of SYCP1 and SYCP3. (D) Meiotic recombination detected by staining for the marker MLH1 to examine the cross-over recombination between two homologous chromosomes. The MLH1 foci could be normally detected on the DSB repair sites in pachytene spermatocyte from *Rnf138^-/-^*. Pictorial data showed representatives of three independent experiments. All bars = 5μm.
